# The age distribution of facial metrics in two large Korean populations

**DOI:** 10.1038/s41598-019-51121-z

**Published:** 2019-10-10

**Authors:** Hae-Young Lee, Seongwon Cha, Hyo-Jeong Ban, In-Young Kim, Bo-Reum Park, Ig-Jae Kim, Kyung-Won Hong

**Affiliations:** 1 Theragenetex Bioinstitute Co. Ltd, Suwon, 16229 Republic of Korea; 2 Future Medicine Division, Korea Institute of Oriental Medicine, Daejeon, 34054 Republic of Korea; 3 Center for Imaging Media Research, Korea Institute of Science and Technology, Seoul, 02792 Republic of Korea

**Keywords:** Ectoderm, Biological metamorphosis, Predictive markers

## Abstract

Growth and alterations in craniofacial morphology have attracted interest in many fields of science, especially physical anthropology, genetics and forensic sciences. We performed an analysis of craniofacial morphology alterations by gender and ageing stage in Korean populations. We studied 15 facial metrics using two large Korean populations (1,926 samples from the Korea Medicine Data Center cohort and 5,643 samples from the Ansan-Ansung cohort). Among the 15 metrics, 12 showed gender differences and tended to change with age. In both of the independent populations, brow ridge height, upper lip height, nasal tip height, and profile nasal length tended to increase with age, whereas outer canthal width, right palpebral fissure height, left palpebral fissure height, right upper lip thickness, left upper lip thickness, nasal tip protrusion, facial base width, and lower facial width tended to decrease. In conclusion, our findings suggest that ageing (past 40 years of age) might affect eye size, nose length, upper lip thickness, and facial width, possibly due to loss of elasticity in the face. Therefore, these facial metric changes could be applied to individual age prediction and aesthetic facial care.

## Introduction

The appearance of the face typically changes in different dimensions and directions with age^1,2^. This phenomenon, known as allometry, is the reason why young faces are distinct from old faces^3^. Growth and alteration in craniofacial morphology have generated interest in many fields of science, especially physical anthropology^4,5^ and genetics^6,7^. Additionally, alternative applications of craniofacial morphology, such as forensic science, have received considerable attention^8^. In the field of Korean Oriental medicine, facial metrics are regarded as representative and reliable characteristics for diagnosing a person’s Sasang constitution^9^. Previously, we performed a two-stage genome-wide association study of facial morphological traits in two large Korean populations (1,926 samples from the Korea Medicine Data Center (KDC) cohort and 5,643 samples from the Ansan-Ansung cohort)^6^.

**Figure 1:**
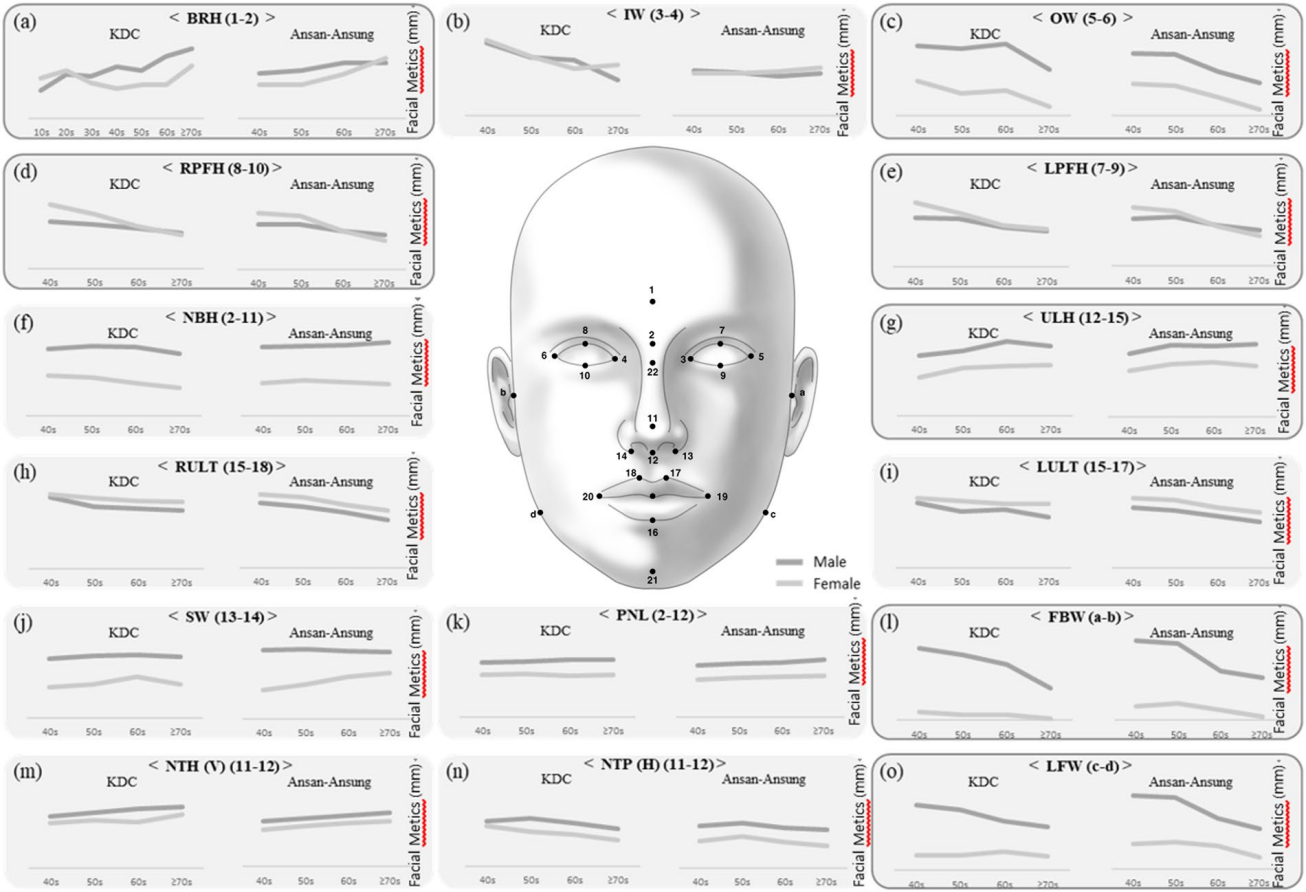
Facial changes from age 40 years to age 70+ years. (**a**) BRH, brow ridge height; (**b**) IW, intercanthal width; (**c**) OW, outer canthal width; (**d**) RPFH, right palpebral fissure height; (**e**) LPFH, left palpebral fissure height; (**f**) NBH, nasal bridge height; (**g**) ULH, upper lip height; (**h**) RULT, right upper lip thickness; (**i**) LULT, left upper lip thickness; (**j**) SW, subnasal width; (**k**) PNL, profile nasal length; (**l**) FBW, facial base width; (**m**) NTH(V), nasal tip height; (**n**) NTP(H), nasal tip protrusion; and (**o**) LFW, lower facial width.

We speculated that the ageing-related trends in each facial metric might be important for human evolution and appearance. In this study, we compared 15 facial metrics – (a) BRH, brow ridge height; (b) IW, intercanthal width; (c) OW, outer canthal width; (d) RPFH, right palpebral fissure height; (e) LPFH, left palpebral fissure height; (f) NBH, nasal bridge height; (g) ULH, upper lip height; (h) RULT, right upper lip thickness; (i) LULT, left upper lip thickness; (j) SW, subnasal width; (k) PNL, profile nasal length; (l) FBW, facial base width; (m) NTH(V), nasal tip height (vertical); (n) NTP(H), nasal tip protrusion (horizontal); and (o) LFW, lower facial width – for each of four age groups (40 s, 50 s, 60 s and over 70 s) using the KDC and Ansan-Ansung cohorts as the replication set (Fig. 1). For example, the brow ridge is the nodule or crest of bone that is situated on the frontal bone of the skull^10^, forming the boundary between the forehead itself and the tops of the eye sockets. Normally, in humans, the ridges arch over each eye, offering mechanical protection^11^. Typically, the arches are more prominent in men than in women and vary between different ethnic groups^12^.

## Results

### Gender differences

The distribution characteristics of 15 facial metrics are described in Table 1 and plotted in Fig. 1 by gender and age group in the KDC and Ansan-Ansung cohorts. The prominent gender differences were identified by the sex differences in the overall sample, as shown in Table 1, and the age distribution of sex differences, as shown in Fig. 1. Among the 15 facial metrics, six metrics (OW, NBH, ULH, SW, FBW, and LFW) appeared to be greater in males than in females in the overall sample comparison in both the KDC and Ansan-Ansung cohorts. In the age distribution of gender differences in Fig. 1, 12 metrics (BRH, OW, NBH, ULH, RPFH, LPFH, SW, PNL, FBW, NTH(V), NTP (H), LFW) showed gender differences. Interestingly, 4 metrics (RPFH, LPFH, RULT, LULT) were larger in females than in males.

**Table 1:** Means and standard deviations of facial metrics in KDC and Ansan-Ansung populations.

Coordinate*	Phenotype	Phenotype description	Sex	Population	Means and standard deviations of facial metrics in each age group (mm)															ANOVA test	Linear regression					
					Total				40			50			60			70		p-value	Adjusted for age			Adjusted by age and BMI		
					N	Mean	SD	N	Mean	SD	N	Mean	SD	N	Mean	SD	N	Mean	SD		Beta	Std. error	Sig.	Beta	Std. error	Sig.
(**a**)	**BRH**	**Brow ridge height**	**Male**	**KDC**	**670**	**29**.**8**	**4**.**3**	**145**	**29**.**8**	**4**	**147**	**29**.**5**	**4**.**6**	**106**	**30**.**6**	**4**.**5**	**73**	**31**.**1**	**4**.**9**	**<0**.**05**	**0**.**10**	**0**.**02**	**<0**.**05**	**0**.**11**	**0**.**02**	**<0**.**05**
				**Ansan-Ansung**	**2541**	**29**.**7**	**4**.**3**	**245**	**29**.**3**	**3**.**8**	**1222**	**29**.**5**	**4**	**658**	**30**.**1**	**4**.**8**	**416**	**30**	**4**.**7**	**<0**.**01**	**0**.**07**	**0**.**01**	**<0**.**001**	**0**.**11**	**0**.**01**	**<0**.**001**
			**Female**	**KDC**	**1164**	**28**.**7**	**4**	**255**	**28**.**2**	**3**.**6**	**250**	**28**.**5**	**3**.**9**	**178**	**28**.**5**	**4**.**4**	**123**	**29**.**9**	**5**	**<0**.**01**	**0**.**12**	**0**.**01**	**<0**.**01**	**0**.**10**	**0**.**01**	**<0**.**01**
				**Ansan-Ansung**	**2721**	**29**	**3**.**9**	**216**	**28**.**4**	**3**.**6**	**1191**	**28**.**4**	**3**.**5**	**808**	**29**.**2**	**3**.**9**	**506**	**30**.**4**	**4**.**3**	**<0**.**001**	**0**.**18**	**0**.**01**	**<0**.**001**	**0**.**18**	**0**.**01**	**<0**.**001**
(b)	IW	Intercanthal width	Male	KDC	708	36.5	3.8	155	36.6	3.6	160	35.9	3.3	113	35.7	4.1	74	34.8	4.5	<0.01	−0.18	0.02	<0.001	−0.17	0.02	<0.001
				Ansan-Ansung	2604	35.6	3.8	248	35.9	3.3	1252	35.7	3.5	674	35.4	3.9	430	35.7	4.5	0.233	−0.03	0.01	0.16	−0.03	0.01	0.13
			Female	KDC	1263	36.4	3.5	275	36.7	3.3	274	35.9	3.6	190	35.3	3.8	131	35.5	3.5	<0.001	−0.15	0.01	<0.001	−0.15	0.01	<0.001
				Ansan-Ansung	2978	35.8	3.5	245	35.6	3	1286	35.6	3.1	871	35.8	3.6	576	36.2	4.2	<0.01	0.05	0.01	<0.05	0.05	0.01	<0.01
(**c**)	**OW**	**Outer canthal width**	**Male**	**KDC**	**716**	**99**.**7**	**7**	**156**	**99**.**7**	**6**.**7**	**160**	**99**.**4**	**6**.**8**	**117**	**99**.**9**	**7**.**2**	**77**	**97**	**8**.**7**	**<0**.**05**	**−0**.**12**	**0**.**03**	**<0**.**01**	**−0**.**10**	**0**.**03**	**<0**.**05**
				**Ansan-Ansung**	**2624**	**99**	**6**.**5**	**249**	**100**.**4**	**5**.**2**	**1254**	**100**.**3**	**5**.**9**	**686**	**97**.**9**	**6**.**6**	**435**	**96**.**4**	**7**.**3**	**<0**.**001**	**−0**.**25**	**0**.**01**	**<0**.**001**	**−0**.**17**	**0**.**01**	**<0**.**001**
			**Female**	**KDC**	**1275**	**95**.**3**	**6**.**2**	**275**	**95**.**8**	**5**.**2**	**274**	**94**.**5**	**6**.**1**	**193**	**94**.**8**	**7**	**140**	**93**	**7**.**3**	**<0**.**001**	**−0**.**14**	**0**.**02**	**<0**.**001**	**−0**.**16**	**0**.**02**	**<0**.**001**
				**Ansan-Ansung**	**2982**	**95**	**5**.**9**	**246**	**96**.**3**	**4**.**9**	**1286**	**96**.**1**	**5**.**3**	**874**	**94**.**6**	**6**	**576**	**92**.**9**	**6**.**8**	**<0**.**001**	**−0**.**21**	**0**.**01**	**<0**.**001**	**−0**.**22**	**0**.**01**	**<0**.**001**
(**d**)	**RPFH**	**Right palpebral fissure height**	**Male**	**KDC**	**717**	**7**.**7**	**1**.**4**	**155**	**7**.**9**	**1**.**4**	**162**	**7**.**7**	**1**.**5**	**117**	**7**.**5**	**1**.**3**	**77**	**7**.**2**	**1**.**5**	**<0**.**001**	**−0**.**17**	**0**.**01**	**<0**.**001**	**−0**.**18**	**0**.**01**	**<0**.**001**
				**Ansan-Ansung**	**2629**	**7**.**5**	**1**.**4**	**249**	**7**.**8**	**1**.**4**	**1254**	**7**.**7**	**1**.**4**	**689**	**7**.**3**	**1**.**5**	**437**	**7**.**1**	**1**.**5**	**<0**.**001**	**−0**.**19**	**0**.**00**	**<0**.**001**	**−0**.**21**	**0**.**00**	**<0**.**001**
			**Female**	**KDC**	**1277**	**8**.**5**	**1**.**7**	**275**	**8**.**9**	**1**.**5**	**276**	**8**.**3**	**1**.**6**	**195**	**7**.**6**	**1**.**5**	**141**	**7**	**1**.**7**	**<0**.**001**	**−0**.**40**	**0**.**00**	**<0**.**001**	**−0**.**39**	**0**.**00**	**<0**.**001**
				**Ansan-Ansung**	**2986**	**7**.**7**	**1**.**6**	**245**	**8**.**5**	**1**.**4**	**1285**	**8**.**3**	**1**.**4**	**876**	**7**.**4**	**1**.**5**	**580**	**6**.**8**	**1**.**4**	**<0**.**001**	**−0**.**40**	**0**.**00**	**<0**.**001**	**−0**.**40**	**0**.**00**	**<0**.**001**
(**e**)	**LPFH**	**Left palpebral fissure height**	**Male**	**KDC**	**715**	**7**.**9**	**1**.**5**	**155**	**8**	**1**.**4**	**162**	**8**	**1**.**6**	**116**	**7**.**4**	**1**.**4**	**75**	**7**.**2**	**1**.**6**	**<0**.**001**	**−0**.**21**	**0**.**01**	**<0**.**001**	**−0**.**22**	**0**.**01**	**<0**.**001**
				**Ansan-Ansung**	**2626**	**7**.**7**	**1**.**5**	**250**	**7**.**9**	**1**.**3**	**1252**	**8**	**1**.**4**	**688**	**7**.**5**	**1**.**5**	**436**	**7**.**2**	**1**.**6**	**<0**.**001**	**−0**.**21**	**0**.**00**	**<0**.**001**	**−0**.**22**	**0**.**00**	**<0**.**001**
			**Female**	**KDC**	**1279**	**8**.**5**	**1**.**7**	**275**	**8**.**9**	**1**.**4**	**276**	**8**.**3**	**1**.**6**	**194**	**7**.**6**	**1**.**7**	**142**	**7**.**3**	**1**.**6**	**<0**.**001**	**−0**.**37**	**0**.**00**	**<0**.**001**	**−0**.**35**	**0**.**00**	**<0**.**001**
				**Ansan-Ansung**	**2977**	**7**.**8**	**1**.**6**	**246**	**8**.**6**	**1**.**5**	**1283**	**8**.**4**	**1**.**5**	**872**	**7**.**4**	**1**.**5**	**576**	**6**.**9**	**1**.**6**	**<0**.**001**	**−0**.**40**	**0**.**00**	**<0**.**001**	**−0**.**40**	**0**.**00**	**<0**.**001**
(f)	NBH	Nasal bridge height	Male	KDC	680	35.8	4.1	150	35.8	3.9	149	36	3.7	110	35.9	4.5	74	35.4	4.4	0.609	−0.02	0.02	0.59	−0.03	0.02	0.48
				Ansan-Ansung	2561	36.1	3.9	246	35.9	3.5	1229	36	3.8	667	36.1	4	419	36.4	4.4	0.112	0.04	0.01	<0.05	0.00	0.01	0.89
			Female	KDC	1210	33.3	3.8	264	33.5	3.4	260	33.3	3.9	185	32.8	3.8	134	32.4	4.3	<0.01	−0.10	0.01	<0.01	−0.09	0.01	<0.05
				Ansan-Ansung	2826	32.9	3.6	230	32.8	3.5	1224	33	3.4	831	32.9	3.7	541	32.8	4	0.372	−0.02	0.01	0.41	−0.01	0.01	0.52
(g)	ULH	Upper lip height	Male	KDC	675	29.5	3	147	29.4	2.9	154	29.8	2.9	112	30.5	3.2	67	30.1	3.6	<0.05	0.11	0.01	<0.05	0.12	0.01	<0.01
				Ansan-Ansung	2524	30.2	2.8	242	29.6	2.5	1226	30.3	2.7	649	30.2	2.9	407	30.4	3.2	0.0612	0.04	0.01	0.05	0.05	0.01	<0.01
			Female	KDC	1252	27.9	2.6	270	27.8	2.3	271	28.5	2.6	190	28.6	2.7	138	28.7	3.1	<0.001	0.11	0.01	<0.001	0.10	0.01	<0.01
				Ansan-Ansung	2941	28.9	2.6	243	28.4	2.2	1279	28.9	2.3	859	29.1	2.7	560	28.8	3	0.245	0.02	0.01	0.29	0.02	0.01	0.33
(h)	RULT	Right upper lip thickness	Male	KDC	677	2.1	0.2	150	2.2	0.2	155	2.1	0.2	108	2.1	0.3	66	2	0.3	<0.001	−0.18	0.00	<0.001	−0.18	0.00	<0.001
				Ansan-Ansung	2476	2.1	0.2	242	2.1	0.2	1216	2.1	0.2	631	2	0.2	387	2	0.3	<0.001	−0.22	0.00	<0.001	−0.22	0.00	<0.001
			Female	KDC	1256	2.2	0.2	272	2.2	0.2	272	2.1	0.2	190	2.1	0.2	133	2.1	0.2	<0.001	−0.13	0.00	<0.001	−0.13	0.00	<0.001
				Ansan-Ansung	2941	2.1	0.2	245	2.2	0.2	1282	2.2	0.2	855	2.1	0.2	559	2.1	0.3	<0.001	−0.25	0.00	<0.001	−0.25	0.00	<0.001
(i)	LULT	Left upper lip thickness	Male	KDC	683	2.1	0.2	152	2.1	0.2	153	2.1	0.2	108	2.1	0.2	69	2	0.3	<0.001	−0.18	0.00	<0.001	−0.18	0.00	<0.001
				Ansan-Ansung	2476	2	0.2	241	2.1	0.2	1212	2.1	0.2	634	2	0.2	389	2	0.3	<0.001	−0.19	0.00	<0.001	−0.19	0.00	<0.001
			Female	KDC	1255	2.2	0.2	272	2.2	0.2	272	2.2	0.2	190	2.1	0.2	132	2.1	0.2	<0.01	−0.12	0.00	<0.001	−0.13	0.00	<0.001
				Ansan-Ansung	2946	2.1	0.2	244	2.2	0.2	1283	2.2	0.2	859	2.1	0.2	560	2	0.3	<0.001	−0.25	0.00	<0.001	−0.25	0.00	<0.001
(j)	SW	Subnasal width	Male	KDC	682	27	3	148	27	2.8	157	27.3	3.3	109	27.4	2.9	72	27.3	2.5	0.437	0.02	0.01	0.60	0.03	0.01	0.46
				Ansan-Ansung	2556	27.9	2.6	244	27.9	2.7	1228	28	2.7	661	27.9	2.6	423	27.7	2.5	0.0811	−0.04	0.01	<0.05	−0.01	0.01	0.51
			Female	KDC	1253	24.6	2.7	269	24.6	2.5	272	24.8	2.6	192	25.5	2.6	136	24.9	2.8	<0.05	0.09	0.01	<0.05	0.06	0.01	0.07
				Ansan-Ansung	2957	25.3	2.4	244	24.5	2.2	1280	25	2.2	867	25.6	2.4	566	26	2.5	<0.001	0.20	0.01	<0.001	0.19	0.00	<0.001
(k)	PNL	Profile nasal length	Male	KDC	685	3.9	0.1	151	3.9	0.1	149	3.9	0.1	111	4	0.1	75	4	0.1	<0.05	0.08	0.00	0.08	0.08	0.00	0.09
				Ansan-Ansung	2566	3.9	0.1	244	3.9	0.1	1235	3.9	0.1	669	3.9	0.1	418	4	0.1	<0.001	0.13	0.00	<0.001	0.12	0.00	<0.001
			Female	KDC	1211	3.9	0.1	263	3.9	0.1	262	3.9	0.1	182	3.9	0.1	135	3.9	0.1	0.556	−0.02	0.00	0.50	−0.02	0.00	0.56
				Ansan-Ansung	2809	3.8	0.1	226	3.8	0.1	1228	3.8	0.1	832	3.8	0.1	523	3.9	0.1	<0.001	0.09	0.00	<0.001	0.09	0.00	<0.001
(l)	FBW	Facial base width	Male	KDC	703	156	9.1	154	157.6	9.1	159	156.7	9.3	117	155.2	8.7	76	151.8	10.4	<0.001	−0.22	0.04	<0.001	−0.20	0.03	<0.001
				Ansan-Ansung	2609	156.4	8.6	246	158.7	7.4	1244	158.2	8.1	681	154.3	8.5	438	153.3	9.4	<0.001	−0.26	0.02	<0.001	−0.13	0.02	<0.001
			Female	KDC	1258	148.1	7.6	271	148.3	6.9	269	147.8	7.4	194	147.8	8.4	142	147.3	9.3	0.237	−0.04	0.02	0.23	−0.09	0.02	<0.01
				Ansan-Ansung	2975	148.8	7.4	245	149	6.8	1281	149.5	6.9	869	148.6	7.5	580	147.6	8.4	<0.001	−0.09	0.02	<0.001	−0.10	0.01	<0.001
(m)	NTH(V)	Nasal tip height	Male	KDC	689	2.7	0.2	148	2.6	0.2	153	2.7	0.1	116	2.7	0.2	76	2.7	0.2	<0.001	0.18	0.00	<0.001	0.19	0.00	<0.001
				Ansan-Ansung	2563	2.6	0.1	245	2.6	0.1	1236	2.6	0.1	666	2.6	0.1	416	2.7	0.2	<0.001	0.15	0.00	<0.001	0.18	0.00	<0.001
			Female	KDC	1202	2.6	0.1	260	2.6	0.1	261	2.6	0.1	182	2.6	0.1	133	2.7	0.2	<0.001	0.15	0.00	<0.001	0.13	0.00	<0.001
				Ansan-Ansung	2775	2.6	0.1	228	2.5	0.1	1215	2.6	0.1	818	2.6	0.1	514	2.6	0.1	<0.001	0.13	0.00	<0.001	0.13	0.00	<0.001
(n)	NTP (H)	Nasal tip protrusion	Male	KDC	682	2.6	0.2	145	2.6	0.2	156	2.6	0.2	111	2.6	0.2	76	2.5	0.2	<0.01	−0.13	0.00	<0.01	−0.13	0.00	<0.01
				Ansan-Ansung	2578	2.6	0.2	248	2.6	0.2	1238	2.6	0.1	669	2.5	0.2	423	2.5	0.2	<0.001	−0.11	0.00	<0.001	−0.12	0.00	<0.001
			Female	KDC	1183	2.5	0.2	260	2.5	0.1	254	2.5	0.2	183	2.5	0.2	124	2.4	0.2	<0.001	−0.24	0.00	<0.001	−0.22	0.00	<0.001
				Ansan-Ansung	2814	2.4	0.2	227	2.4	0.2	1229	2.5	0.2	829	2.4	0.2	529	2.4	0.2	<0.001	−0.15	0.00	<0.001	−0.15	0.00	<0.001
(o)	LFW	Lower facial width	Male	KDC	697	136.5	10.3	153	138.7	10	156	138	9.5	116	135.6	9.9	74	134.5	11.5	<0.001	−0.19	0.04	<0.001	−0.16	0.04	<0.001
				Ansan-Ansung	2592	138.1	9.7	246	140.5	8.8	1245	140.1	9.2	674	136.1	9.7	427	134	9.9	<0.001	−0.26	0.02	<0.001	−0.13	0.02	<0.001
			Female	KDC	1256	128.4	8.7	270	128.9	8.3	269	129	8.3	193	129.6	9.2	142	128.7	9.7	0.805	0.00	0.03	0.96	−0.07	0.02	<0.05
				Ansan-Ansung	2971	130.5	8	246	131	7.7	1280	131.2	7.5	868	130.5	8.1	577	128.4	8.7	<0.001	−0.12	0.02	<0.001	−0.13	0.01	<0.001

### Age group comparison

Age group differences were analysed by Student’s t-tests comparing the 40 s age group to the 50 s, 60 s and 70 s age groups. BRH, ULH, and NTH (v) are slightly increased in the 60 s age group in both cohorts and in both genders (Fig. 1a,g,m). OW, RULT, LULT, and NTP (H) were slightly decreased in the 60 s age group in both cohorts and in both genders (Fig. 1c,h,I,n). RPFH and LPFH showed a considerably decreased pattern in both cohorts and in both genders, and the pattern was prominent in females in both cohorts (Fig. 1d,e). Although FBW and LFW showed a considerably decreased pattern in both cohorts and in both genders, the pattern was prominent in males in both cohorts (Fig. 1l,o). PNL showed a slight increase in both cohorts, but only in males (Fig. 1k). IW, NBH, and SW seemed to have no clear pattern (Fig. 1b,f,j).

To understand in greater detail the effect of ageing on facial metrics, we constructed linear regression models with age and/or body mass index as covariates. We identified a significant linear increasing tendency of BRH and NTP and a decreasing tendency of OW, RPFH, LPFH, RULT, LULT, NTH, and NTP in both cohorts and in both sexes (Table 1).

### Ethnic comparison of the human orbital region

**Figure 2:**
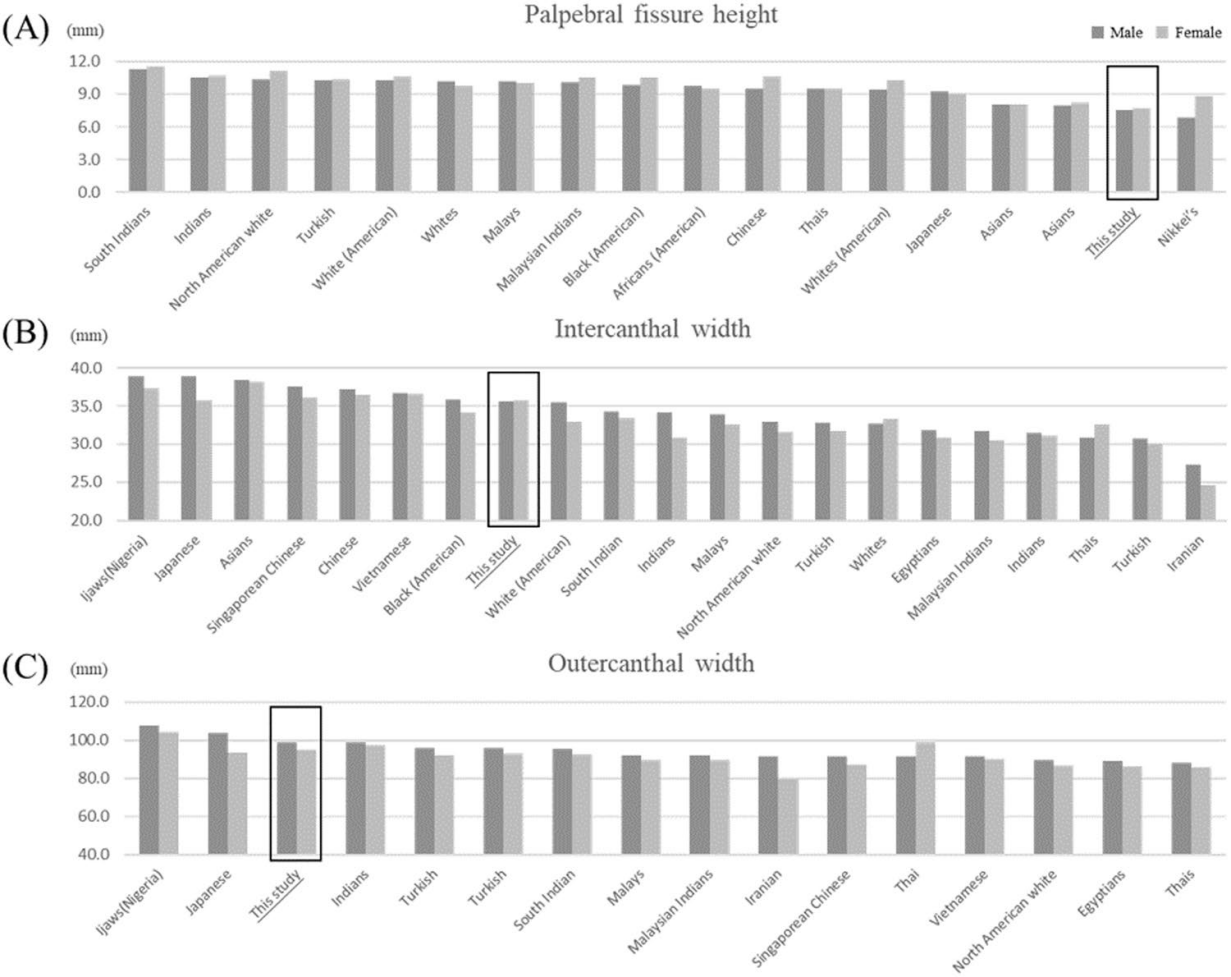
Ethnic comparison of the human orbital region. (**A**) Palpebral fissure height, (**B**) intercanthal width, and (**C**) outer canthal width. The data on ethnicities other than Korean were obtained from Vasanthakumar *et al*.^14^.

Facial metrics in the orbital region, including PFH, IW, and OW, were compared between Koreans and other ethnicities (Fig. 2). RPFH and LPFH were relatively low among non-Asians relative to Asians (Fig. 2A). The Korean subjects had a large OW, ranking between those of Japanese and Indian individuals, whereas the IW was of Koreans was smaller than those of other Asian populations, such as Japanese and Chinese (Fig. 2B,C). Interestingly, the gender difference in IW was no more prominent in Koreans than in other ethnic groups.

## Discussion

In this study, we analysed 15 facial metrics for gender differences and for ageing-related changes in two large Korean cohorts. Among these 15 metrics, 12 showed gender differences and tendencies toward age-related change. Increasing tendencies were observed in BRH, ULH, NTH, and PNL, and decreasing tendencies were observed in OW, RPFH, LPFH, RULT, LULT, NTP, FBW, and LFW. The decreasing tendencies were not changed even when adjusted for body mass index, indicating that some other structures under the skin (such as collagen and muscle) were reduced in the aged population.

In the orbital region, BRH showed a tendency to increase with age in both the KDC and Ansan-Ansung cohorts. The BRH is the nodule or crest of bone situated on the frontal bone of the skull^10^; this ridge separates the forehead itself from the tops of the eye sockets. Normally, in humans, the ridges arch over each eye, offering mechanical protection^11^. Typically, the arches are more prominent in men than in women and vary between different ethnic groups^12^.

“RPFH” and “LPFH” are abbreviations for the right and left palpebral fissure height. The palpebral fissure is the elliptic space between the medial and lateral canthi of the two open eyelids. There are many studies of palpebral fissure height; this metric is approximately 10 mm in adults and is smaller in East Asian populations than in white populations^13,14^. The gender differences in palpebral fissure height have been well summarized in reports on a South Indian population^14^. The bilateral orbital region, which is part of the upper face, acts as a key determinant of the perception of facial attractiveness, youthfulness and health^14^. Our comparison of palpebral fissure height implies that a very small PFH is a typical Korean facial characteristic (Fig. 2A). IW did not show any alteration with age, but OW showed a prominent decreasing pattern. The ethnic comparison of Fig. 2B,C showed that our study group had a shorter IW than Japanese or Chinese individuals, but the gender difference was no more prominent in Koreans than in other ethnic groups. In contrast, the OW of our study population was relatively large and was similar to that of the Japanese population. Therefore, the characteristics of the orbital region of Koreans are a small palpebral height and a wide canthal region. In the nasal area, the NTH slightly increased with age, and the NTP slightly decreased with age, but the NBH did not show any tendency to change. Additionally, the profile nasal length increased slightly with ageing only in males. The nasal tip is a challenging part of the nose for plastic surgeons because of variations in the anatomy of the lower lateral cartilage^15^. Therefore, our results help to clarify the direction of surgical correction for nasal tip deformities.

In the orthodontic region, the ULH slightly increased with ageing, and the RULT and LULT showed slight decreases with ageing. The upper lip is an important attractive point for the smile. According to Hulsey, the “smile is one of the most effective means by which people convey their emotions”^16^. The ULH is affected by the gingival margin of the upper central incisors and influences the attractiveness of a smile^16^. Generally, a shorter ULH indicates a higher smile line. On average, the smile line was found to be 1.5 mm higher in women than in men^17^; similarly, in the current study, the ULH was greater in males than in women, indicating a shorter smile line in women (see Table 1g). Although the concept of beauty has changed throughout the centuries, the thickness of the upper lip has always been a subject of interest and has importance in every culture^18^. These results may be beneficial to forensic anthropologists, plastic and reconstructive surgeons, and orthodontists.

Regarding overall facial width, both FBW and LFW slightly decreased with ageing, and the tendency was more prominent in male than female subjects. Facial width is reportedly associated with testosterone levels in males^19^. Therefore, the decrease in testosterone in elderly males may decrease the facial width.

## Conclusion

Our study found that 12 of 15 facial metrics changed with age in subjects of both genders. The changes in eye size, nose length, upper lip thickness, and facial width might be general trends in Koreans because of the consistent tendencies in two large independent cohorts. Changes in facial metrics could serve as indicators to predict age from photographs and would be helpful for aesthetic facial care. To our knowledge, there are very few studies on the alteration of facial metrics with ageing, particularly in groups over 40 years old. Moreover, our study did not analyse the facial ageing of other ethnic populations. Therefore, we hope that the currents results will be validated in other ethnic groups and applied to diverse fields.

## Materials and Methods

### Study participants

A total of 1,926 participants in the KDC cohort were recruited from 19 sites (Korean Oriental Medical Clinics) between 2007 and 2010^6^, and a total of 5,643 participants in the Ansan-Ansung cohorts were recruited from two regions in southern Korea from 2009 to 2012 for the Korean Genome and Epidemiology Study (KoGES)^6,20^ (Table 2). The subjects were photographed with a neutral expression in both frontal and profile views under the following standard conditions: the hair should be pulled back with a hair band; the centre points of the two pupils should be horizontally aligned, as should the upper auricular perimeters; and a ruler should be placed approximately 10 mm below the chin to convert pixels into millimetres. All participants provided written informed consent to participate in the study. This protocol was approved by the Korea Institute of Oriental Medicine Institutional Review Board (I-0910/02-001), and all research was performed in accordance with the relevant guidelines/regulations.

**Table 2:** Study Population Characteristics.

Phenotypes	Population	Total					Male		Female	
		*N*	*Mean*	*SD*	*N*	*Mean*	*SD*	*N*	*Mean*	*SD*
BMI (kg/m^2^)	KDC	1,408	23.8	3.1	724	24.0	3.1	1,284	23.1	3.3
	Ansan-Ansung	5,643	24.4	3.1	2,648	24.3	2.9	2,995	24.6	3.2
Age (years)	KDC	1,408	57.1	10.8	724	56.9	10.5	1,284	57.2	11.0
	Ansan-Ansung	5,643	60.4	8.5	2,648	59.9	8.4	2,995	60.9	8.6

### Craniofacial measurements

Detailed descriptions of candidate feature variables and the corresponding measurement methods have been provided in a previous report^6,9^. Briefly, the facial variables were limited to those that could be easily quantified. Facial feature points in frontal and lateral images were automatically extracted by detecting and analysing the face, eyes, nose, mouth, and contours via an in-house program in Visual Studio C++ using OpenCV (Open Source Computer Vision Library). The positions of the extracted points were confirmed by a well-trained operator (accuracy: 98.8% on average). Fifteen facial metrics, each defined by the distance between two facial points, were derived from the photographs by converting pixels into millimetres using MATLAB software^21^.

For statistical analysis, 5 severely skewed facial variables, including PNL, NTH(V), NTP(H), RULT, and LULT, were ln transformed. We removed the outliers, which were defined using the first and third quartiles and the interquartile range of each facial variable. In each facial variable, measurements below the first quartile – 2.0 × interquartile range or above the third quartile + 2.0 × interquartile range were defined as outliers and excluded.

### Statistical analysis

For each facial variable, the mean length was compared between age groups (40 s vs 50 s, 60 s or 70+) using Student’s t-test. Additionally, the mean lengths of male and female facial variables were plotted (Fig. 1) for both the KDC and Ansan-Ansung cohorts. We used the following criteria to identify phenotypes that tended to change with age: the mean underwent a significant gradual increase or decrease (p < 0.05), and the pattern appeared to be similar between the KDC and Ansan-Ansung cohorts.
